# Carnivore Translocations and Conservation: Insights from Population Models and Field Data for Fishers (*Martes pennanti*)

**DOI:** 10.1371/journal.pone.0032726

**Published:** 2012-03-27

**Authors:** Jeffrey C. Lewis, Roger A. Powell, William J. Zielinski

**Affiliations:** 1 Washington Department of Fish and Wildlife, Olympia, Washington, United States of America; 2 Department of Biology, North Carolina State University, Raleigh, North Carolina, United States of America; 3 Unite States Department of Agriculture Forest Service, Pacific Southwest Research Station, Arcata, California, United States of America; University of Alberta, Canada

## Abstract

Translocations are frequently used to restore extirpated carnivore populations. Understanding the factors that influence translocation success is important because carnivore translocations can be time consuming, expensive, and controversial. Using population viability software, we modeled reintroductions of the fisher, a candidate for endangered or threatened status in the Pacific states of the US. Our model predicts that the most important factor influencing successful re-establishment of a fisher population is the number of adult females reintroduced (provided some males are also released). Data from 38 translocations of fishers in North America, including 30 reintroductions, 5 augmentations and 3 introductions, show that the number of females released was, indeed, a good predictor of success but that the number of males released, geographic region and proximity of the source population to the release site were also important predictors. The contradiction between model and data regarding males may relate to the assumption in the model that all males are equally good breeders. We hypothesize that many males may need to be released to insure a sufficient number of good breeders are included, probably large males. Seventy-seven percent of reintroductions with known outcomes (success or failure) succeeded; all 5 augmentations succeeded; but none of the 3 introductions succeeded. Reintroductions were instrumental in reestablishing fisher populations within their historical range and expanding the range from its most-contracted state (43% of the historical range) to its current state (68% of the historical range). To increase the likelihood of translocation success, we recommend that managers: 1) release as many fishers as possible, 2) release more females than males (55–60% females) when possible, 3) release as many adults as possible, especially large males, 4) release fishers from a nearby source population, 5) conduct a formal feasibility assessment, and 6) develop a comprehensive implementation plan that includes an active monitoring program.

## Introduction

Since the settlement of Europeans in North America, the ranges of many of the continent's carnivores have contracted significantly (e.g., black-footed ferrets [*Mustela nigripes*], wolves [*Canis lupus*], Canada lynxes [*Lynx canadensis*], wolverines [*Gulo gulo*], fishers [*Martes pennanti*], grizzly bears [*Ursus arctos*]; [1]–[6]). Translocations – the intentional transport and release of animals to reestablish, augment or introduce a population – have been used in attempts to recover extirpated or depleted populations. Translocations, however, are not always successful [7]–[9]. Carnivore translocations can be expensive, time-consuming and controversial; and their success may depend upon adequate planning, expertise, organization, and cooperation [7], [10]. Translocations that fail, even those that are well-planned and well-executed, can erode the support necessary to continue restoration efforts for imperiled species [11]. The International Union for Conservation of Nature (IUCN) has provided guidelines for translocations [10], [12] and several sources provide specific recommendations and cautions for carnivore translocations [7]–[9]. However, specific recommendations are lacking for many species, and wildlife managers may have little to guide them when developing a translocation program. Understanding factors that are associated with translocation success is critically important for developing adequate feasibility studies and effective implementation plans, yet such factors are often unknown.

Having recommendations would be especially valuable for the fisher, which is a candidate for federal endangered status in the Pacific states (California, Oregon, Washington) [13], is listed as an endangered species in Washington [14], and is a target species for recovery efforts in the Pacific states [15], [16]. While fishers have been translocated successfully to a number of locations in eastern and central North America, many translocations in western North America failed to re-establish populations [1], [3], [14], [15], [17].

The fisher is a mid-sized carnivore in the weasel family (Mustelidae) that occurs only in the temperate and boreal forests of North America [1]. Since the mid-1800s, the fisher's geographic distribution contracted substantially [18], due probably to historical over-trapping, non-compensatory mortality from predator-control campaigns and incidental trapping [19], habitat loss and fragmentation [1], and climatic changes in eastern North America associated with the Little Ice Age [20]. The extremely high prices paid for prime fisher pelts (up to $350/pelt in the early 1900s) [21]–[24], their vulnerability to trapping [1], and a lack of harvest regulations resulted in unsustainable exploitation of many fisher populations. By the mid-1900s, despite protections established for fisher populations throughout much of their historical range (i.e., range prior to European settlement), many populations did not recover. Moreover, the loss and fragmentation of structurally complex forests due to timber harvest, human development, changes in fire regimes, and climate change likely exacerbated population declines and impeded or prevented the recovery of many populations.

When the fisher's range was most contracted, large portions of its historical range in the US and southern Canada were unoccupied [25]. By the early 1900s, small populations of fishers persisted in only 6 locations in the US: northwestern California and southwestern Oregon; the southern Sierra Nevada; the Bitterroot Mountains in north-central Idaho and west-central Montana; the Big Bog area of northern Minnesota; Adirondack Park in northern New York; and the White Mountains and Moosehead Plateau in northern New Hampshire and northwestern Maine [1], [3], [25]–[33].

During the mid-1900s, many resource management agencies and timber companies suffered significant tree losses from unusually large porcupine (*Erithizon dorsatum*) populations, which they attributed to the absence of fishers. This prompted many wildlife and forest management agencies to reintroduce fishers to restore an effective predator of porcupines and a valuable furbearer [29], [34]–[38]. Although many of these reintroduction efforts and their outcomes have been reported in the literature [1], [3], [17], [36], [39]–[45], they have not been evaluated to identify factors that influence the success of fisher translocations.

In this paper we: (1) present a population model for fisher reintroductions and use the model to predict factors that influence translocation success; we also use the model to evaluate the population-level effects of removing fishers from a source area for translocation; (2) summarize data from actual fisher translocations and use those data, combined with demographic data from the literature, to evaluate factors that may influence translocation success; (3) use data from actual translocations to test the predictions of the population model; (4) evaluate the contributions of translocations to fisher conservation; and (5) provide managers with recommendations to increase translocation success.

## Methods

We define translocation as the intentional transport and release of animals to reestablish, augment or introduce a population in the wild. We define a reintroduction as an attempt to reestablish a population where one no longer exists within a species' historical range, an augmentation as adding individuals to an existing population, and an introduction as an attempt to establish a population outside the species' historical range [12], [46]. We considered translocations successful if the target population was reestablished (i.e., for reintroductions), established (introductions) or growing (augmentations), as determined by the resource agency responsible or as documented in the literature. We concluded that reintroductions or introductions had failed when active monitoring or incidental observations (e.g., road kills, sightings, trapped fishers) indicated a consistent lack of detections of fishers (or lack of population growth, for augmentations) in the vicinity of the release area, as documented in the literature or in unpublished reports.

### Population Model for Fishers

We used the population simulation program *VORTEX* [47] to model fisher populations because it allowed us to develop models for both reintroduced and source populations. We used *VORTEX* to explore characteristics of reintroduction programs that might lead to success or failure, assuming that release sites had adequate habitat. We incorporated the values for demographic parameters shown in Table 1, taking values from Powell's review [1] and data for survival reported by Raine [48]. We used these values to develop both a baseline population model and alternative models that represented a number of reintroduction and source population scenarios.

**Table 1:** Values for demographic parameters and characteristics of the model source fisher population.^1^

		Elasticity	
Demographic Variable	Value (± SD)	−10%	+10%
Starting population size, *N_0_*	1000	−4	2
Carrying capacity, *K*	2000±250	−7	8
Mean litter size	2.0±1.0	−23	3
Age (yr) first reproduction	2	—2	—2
Exponent for density dependence, *B*	16	0	0
Exponent for Allee Effect, *A*	0.5	0	0
Survival rates			
Juveniles (age 0–1)^3^	35±25%	−26	6
Yearlings (age 1–2)	75±20%	−9	7
Adults (age ≥2)	88±20%	−15	10
Reproduction after logging	50%	−4	8
Survival after logging	75%	—2	—2
Local subpopulations (N = 50; and timber harvest/100 yrs)			
On private lands	25 (2 harvests/100 yrs)		
On USFS and BLM lands managed for timber	18 (1 harvest/100 yrs)		
On USFS and BLM protected lands	7 (no harvest)		

1 Simulations were run 100 times for 100 years using *VORTEX* with stochastic variation as indicated by standard deviations (SD). Elasticity indexes the change in the viability index (1 minus the probability of extinction) when input variables are changed by ±10%.

2 Elasticity values not calculated.

3 Juveniles constituted ∼45% of the population.

Next, we simulated environmental conditions for a source population occupying a landscape partitioned into a grid of 50 contiguous subsites (the maximum number allowed by *VORTEX*) that we envisioned as hexagons covering the landscape. Each hexagon contained habitat capable of supporting 20, 40 or more fishers, depending on the model conditions (hexagon capacity = *K*/50). Across their range, fishers experience distinctly different habitat configurations at the landscape scale. In the West, fishers occupy forested landscapes that are predominantly a patchwork of private and federal ownership (16). Therefore, we designated 25 hexagons as private land managed for timber with the probability of 2 harvests that removed critical habitat each 100 years, 18 as land managed by USDA Forest Service or USDI Bureau of Land Management for multiple uses with 1 harvest that removed critical habitat each 100 years, and 7 as protected land with no history of harvest. During the year after harvest, we reduced reproduction by 50% and survival by 15% in all hexagons. Because *VORTEX* allows changes for only 1 year after significant events, we exaggerated the reduction of reproduction and survival in the year following harvest to gain a long-term effect. We assigned a 5% probability of a dispersing juvenile moving to each adjacent hexagon, and a 3% probability of moving to other hexagons.

The fisher population in northwestern California is currently being used as the source population for a reintroduction in the northern Sierra Nevada of California. This source population is more restricted in area than the populations in British Columbia, Minnesota and New York, which have commonly been used as source populations for reintroductions. If removing fishers has an effect on a source population, the effects should be most pronounced on smaller source populations. Therefore, we set the carrying capacity (*K*) for our simulated source population at 2000, a number that is roughly modal for unpublished estimates (no published estimates exist) for the population in northwestern California. We modeled a population that was not subject to trapping or hunting mortality.

We calculated elasticity values for demographic parameters in Table 1 by varying the baseline values ±10%. We did not vary values for sex ratio or age of first reproduction because they were considered the least variable demographic characteristics [1], [49]. We ran each set of values for each variable 100 times and used 1 minus the mean probability of extinction as an *index* of population viability (*i.e.*, high index values indicate a high probability of viability), not as a direct estimate.

To evaluate the potential population-level effects of removing fishers from the source population, we removed 20 fishers from the source population for each of 2, 3, 5 or 8 years, removing either 5 fishers from each of 4 different hexagons each year or 1 fisher from each of 20 hexagons. To increase the effects of losing reproductive females from the source population, we removed adults only and removed 3 females for every 2 males.

To compare the effectiveness of different reintroduction approaches, we simulated the release of fishers onto an empty landscape of 50 hexagons in several ways, including: 1) the release of 20 fishers (5 in each of 4 hexagons) for each of 2, 3, 5 or 8 years, releasing fishers into new hexagons each year; 2) releasing 20 to 160 fishers, all released in 1 year (5 per hexagon); and 3) releasing 80 fishers all in 1 year into 1, 2, 3 … 20 hexagons. We assumed that the simulated reintroductions would occur on a landscape with 80% federal land (40 hexagons) and 20% private land (10 hexagons), which is consistent with ownership patterns where fishers were recently reintroduced in Washington and California [15], [50]. Consistent with the simulated source population, the simulated reintroduction area was subject to timber harvests. To explore the effects of sex and age ratios on potential reintroduction success, we varied the sex ratio from 4∶1 (M∶F) to 1∶4, and varied the number of juveniles released from 0 to 3 in each group of 5 fishers released in a hexagon. We assumed that the reintroduced population of fishers would initially occupy a smaller area than the source population, and therefore set *K* at 1,000. Values for other variables were the same as those for the source population. For this modeling exercise, we considered a reintroduction successful if the population persisted for 100 years.

### Actual Fisher Translocations

We compiled information on fisher translocations from the scientific and popular literature; theses and dissertations; agency databases, files, and archives; and interviews with individuals that participated in or had knowledge of the translocations, substantially expanding the information reported previously [1], [17], [51]. We documented the following characteristics of each translocation: (1) translocation type (reintroduction, augmentation or introduction), (2) location (state or province and specific release sites), (3) outcome (success or failure), (4) source population (state or province), (5) purpose of translocation, and (6) years of initiation and completion. Additional factors that could influence translocation success are presented in Table 2.

**Table 2:** General characteristics (variables) of fisher translocations that could influence translocation success.

Variables that could influence translocation success	Variable name	*VORTEX*	Data
Number of fishers released	Number of fishers	Yes	Yes
Number of release sites	Number of sites	Yes	Yes
Number of years fishers released	Number of years	Yes	Yes
Sex ratio of released fishers	Sex-ratio	Yes	Yes
Feasibility assessment prior to release	Feasibility	No	Yes
Genetic diversity of source population (number sources)	Diversity	No	Yes
Genetic relatedness to source population (proximity)	Relatedness	No	Yes
Monitoring post-release	Monitor	No	Yes
Protection from fur-trapping for fishers specifically	Protect1	No	Yes
Protection from incidental fur-trapping	Protect2	No	Yes
Region (Eastern *vs* Western North America^1^)	Region	No	Yes
Season of release	Season	No	Yes
Type of release (hard versus soft)	Type	No	Yes
Ownership of lands where fishers released	Owner	No	No
Trapping re-established following translocation	Trapping	No	No

Four variables were included in our *VORTEX* simulations and data for 13 of these variables were available from actual translocations.

1 106° West Longitude, chosen because it is midway within a gap between translocations that occurred in eastern versus western North America (see Figure 6).

To evaluate the extent to which successful translocations may have contributed to recent range expansions, we overlaid the locations of release sites on 3 different depictions of the fisher's geographic distribution: the historical range, the range at its most geographically contracted state (hereafter, most-contracted range), and the current range. We developed these 3 range maps based on previously reported ranges [1], [18], [28], [52]–[54]); data from museum collections, published literature, and unpublished reports; and interviews with agency personnel and local experts.

We considered translocations to be independent if they were separated by ≥200 km (163 km is the largest post-release movement documented; [51]). Translocations were also considered independent if they occurred within 200 km of each other but the earlier translocation failed.

### Test of Population Model Predictions

We used data from actual reintroductions to test the predictions of our *VORTEX* model and to determine if we obtained similar results for reintroduction success from these 2 independent approaches (*VORTEX* model vs. data from actual reintroductions). Before testing hypotheses we derived from *VORTEX* model results, we tested for independence of the variables from actual reintroductions using Pearson's correlation coefficient (α = 0.05). Using data from actual reintroductions, we calculated Akaike's Information Criterion adjusted for small sample sizes (AIC_C_; [55]) and evaluated the hypotheses we derived from the *VORTEX* model results. We calculated likelihood values for AIC_C_ using Proc GENMOD in SAS (SAS 9.2; www.sas.com), and retained all hypotheses with ΔAIC_C_≤2.0.

Weights of released fishers are included in some translocation reports, but no dependable age or maturity data exist for any of the reintroductions with a known outcome. Sex-ratio data, however, exist for the majority of reintroductions. Therefore, we first evaluated the strengths of the following hypotheses to explain reintroduction success.

(1) Number of fishers released, as a substitute for number of adult females, allowing us to use data for all reintroductions. Our *VORTEX* model results suggested that the probability of a successful reintroduction should increase with the number of adult females released.

(2) Number of females released, as a substitute for number of adult females.

(3) Number of males released, because our *VORTEX* model results suggested, perhaps counter-intuitively, that number of adult males released should have no effect on reintroduction success.

(4) Number of release sites, because our *VORTEX* model results suggested that the probability of a successful reintroduction should increase with the number of release sites used.

(5) Number of release years (controlled for total numbers of fishers released), because our *VORTEX* model results suggested, again perhaps counter-intuitively, that the number of years over which a given number of fishers was released should have no effect on reintroduction success.

(6–14) The combinations of variables in (1), (2) or (3) with those in (4) and (5).

For the hypotheses with ΔAIC_C_≤2.0, we evaluated additional factors (variables) from Table 2, taken one at a time, again using AIC_C_ to rank the performance of our hypotheses. Genetic diversity of the source population and genetic relatedness of the source population to the original population were unknown for most translocations. We used the number of states and provinces that provided source populations (which was known for all translocations) as an index of genetic diversity, and we used proximity of source population to the release site (same part of continent = near, otherwise far) as an index of genetic relatedness. We started with “Region” and noted that this variable has such a large effect on AIC_C_ values that we evaluated all other single variables along with the “Region” variable.

## Results

### Population Models for the Fisher

*VORTEX* simulations of the baseline population model (i.e., no fishers removed) had a 95% viability index. Baseline simulations also indicated that juvenile survival and litter size had the greatest elasticity values, suggesting that variation in these variables had the greatest potential to affect the viability index (Table 1). Adult survival had intermediate elasticity values; variation in other variables had little effect on the index.

To account for the possibility that our estimates of the present population size or carrying capacity for fishers in northwestern California were overestimates, we also ran simulations with each of these variables reduced to half of its original value. Halving the initial population size or halving *K* decreased the viability index by about 1%, which is a smaller effect than caused by 10% changes in juvenile survival or litter size.

Model results for the source population indicated that the removal of fishers from the population had little effect on the viability index (Table 3). The index dropped <5% when 20 fishers (5 each from 4 different hexagons each year) were removed from the model population for each of 8 years; removals of 1 fisher from each of 20 hexagons for 3 years had no effect on the index.

**Table 3:** Predicted effects of removing fishers for 3, 5, or 8 years on the viability of a source population.^1^

Removal Scenario			Decrease in Viability Index
Number fishers removed from each hexagon	Number hexagons	Number years	
1	20	3	0%
1	20	5	3%
5	4	3	2%
5	4	5	0%
5	4	8	1%

1 The source population had a carrying capacity (K) of 2000; viability declines were predicted by 100 runs of *VORTEX* for each set of values for variables. Each of 50 hexagons was modeled to determine landscape effects; simulated animals could move among the hexagons.

**Figure 1:**
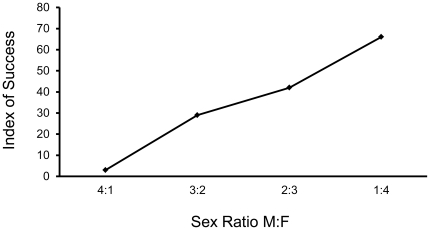
Predicted relationship between sex ratio for fishers that are released and reintroduction success. The *VORTEX* model included stochastic variation and was run for 100 years. Twenty fishers were modeled to be released in each of 5 years in 4 groups of 5, with each group released into a different site.

**Figure 2:**
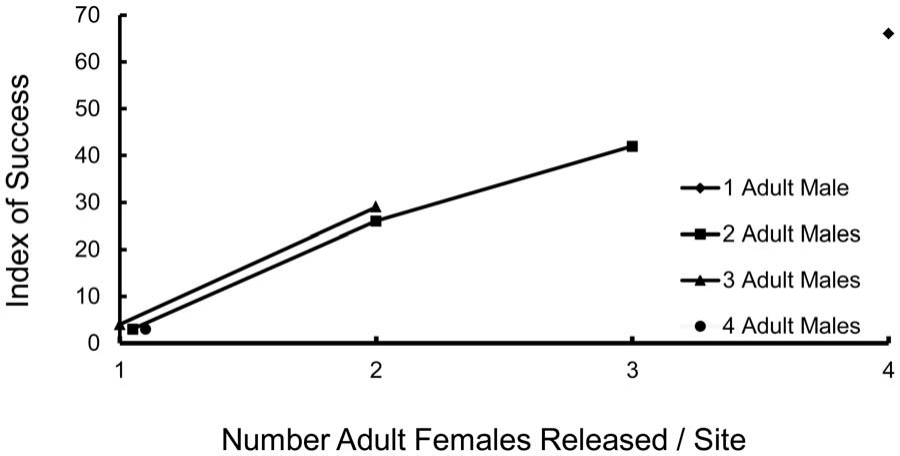
Predicted relationship between numbers of adult female and adult male fishers released and reintroduction success. Fishers were released at each of 4 subsites in each of 5 years. No juvenile fishers were released in these scenarios. The *VORTEX* model included stochastic variation and was run for 100 years. Twenty fishers were modeled to be released each of 5 years in 4 groups of 5, with each group released into a different site.

**Figure 3:**
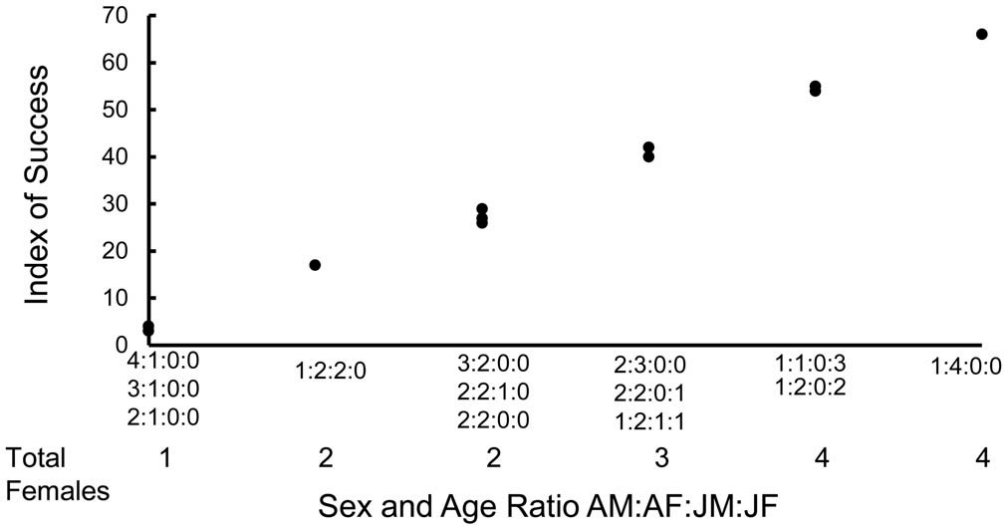
Predicted relationship between numbers of female and male fishers released, including juveniles, and reintroduction success. Fishers were released at each of 4 subsites in each of 5 years. The *VORTEX* model included stochastic variation and was run for 100 years. Twenty fishers were modeled to be released each of 5 years in 4 groups of 5, with each group released into a different site.

**Figure 4:**
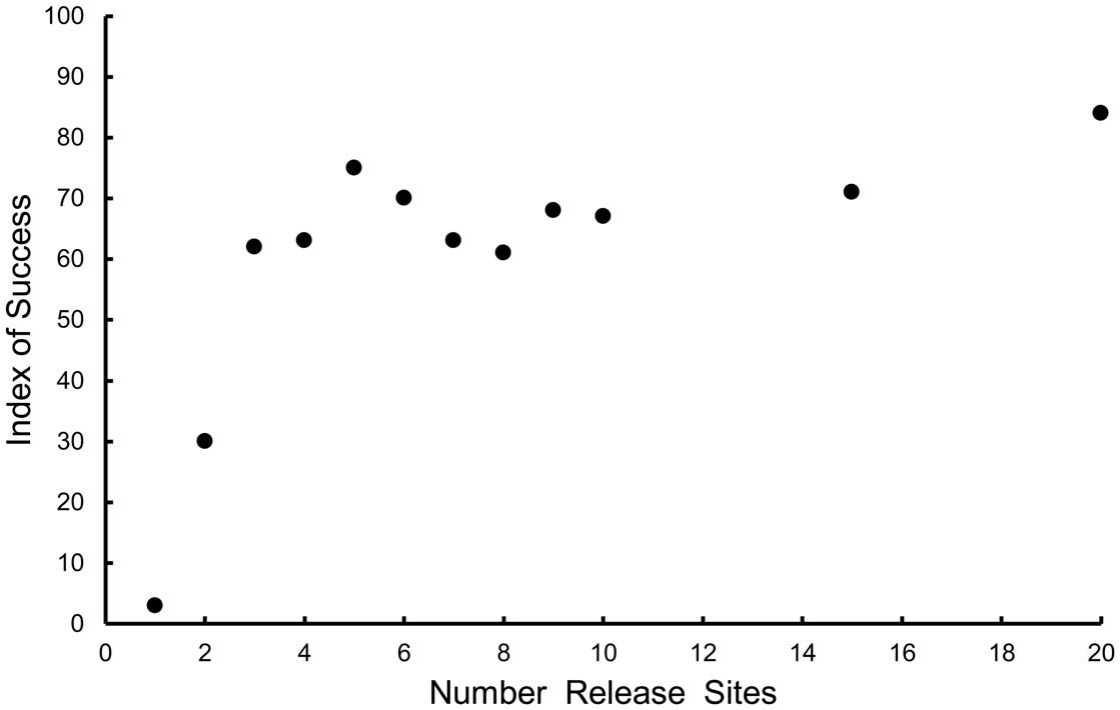
Predicted relationship between the number of release sites for a translocation and reintroduction success. Points represent mean values for 100 simulations.

**Figure 5:**
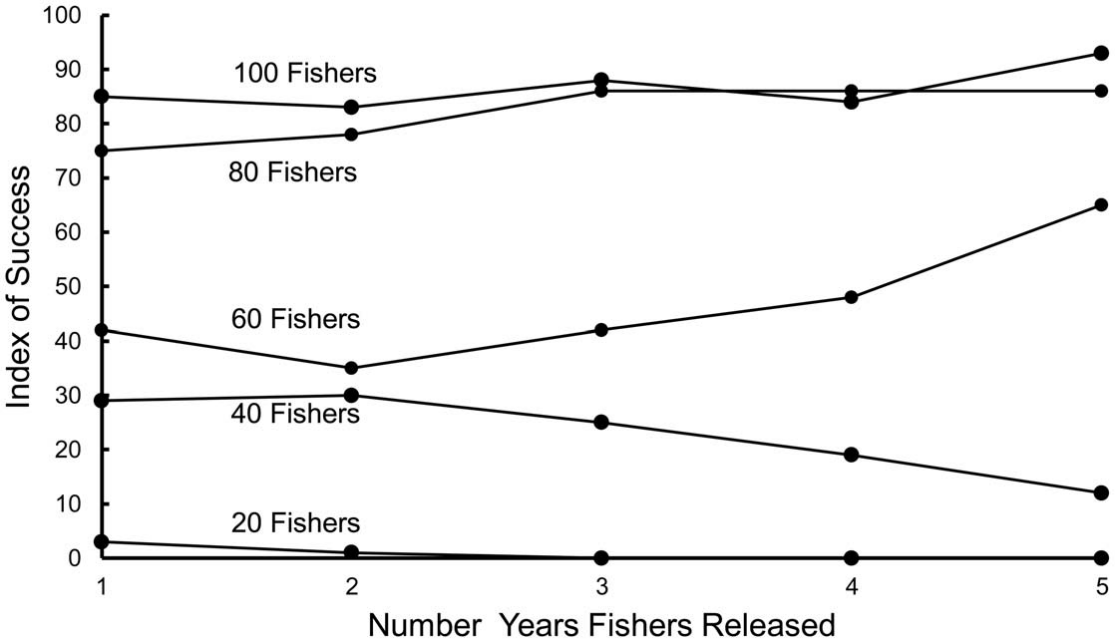
Predicted relationship between the number of years fishers are released and reintroduction success. The index of success was predicted for a fixed number of fishers released all in one year or released over 2, 3, 4 or 5 years (e.g., 60 fishers released in 1 year; 30 in each of 2 years; up to 12 in each of 5 years). Points represent mean values for 100 simulations.

The index of success for simulated populations of reintroduced fishers varied considerably with the reintroduction scenario (Table 4, Figures 1, 2, 3, 4, 5). The index increased with the number of adult females in the release population as long as 1 adult male was also included (Table 4, Figures 1, 2, 3), with the exception that releasing 2 adult females and 2 juvenile females was equivalent to releasing 1 adult female and 3 juvenile females (Figure 3). Releasing juvenile females and males, or more than 1 adult male, reduced reintroduction success by limiting the number of adult females released (Figure 3). The index of success increased with the number of release sites (Figure 4) but the number of years used to release a fixed number of fishers had no effect on success (Figure 5).

**Table 4:** Predicted index of successful reintroduction of fishers from 100 runs of *VORTEX* for differing founder population compositions.

Reintroduction Scenario: number released at each of 5 release sites each year					Index of Success
Adult females	Adult males	Juvenile females	Juvenile males	Number of release years	
3	2	0	0	2	2
3	2	0	0	3	20
3	2	0	0	5	42
3	2	0	0	8	82
1	4	0	0	5	3
1	3	0	0	5	4
1	2	0	0	5	3
2	3	0	0	5	29
2	2	0	0	5	26
4	1	0	0	5	66
2	2	1	0	5	40
2	2	0	1	5	27
2	1	1	1	5	40
2	1	2	0	5	54
2	1	0	2	5	17
1	1	3	0	5	55

### Actual Fisher Translocations and Subsequent Range Expansions

**Figure 6:**
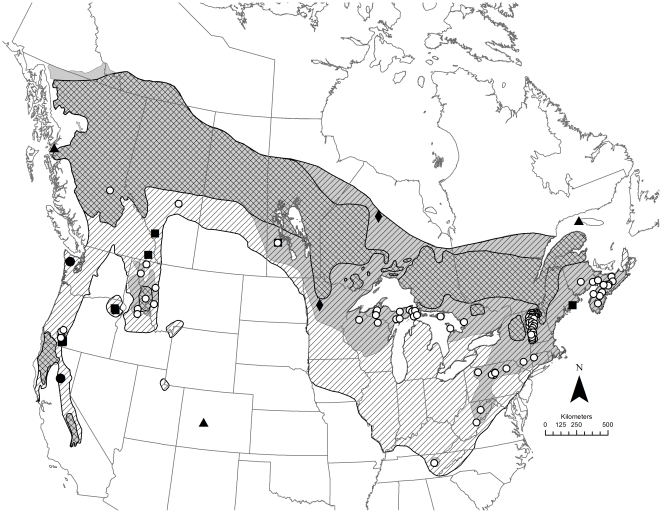
Locations of translocations in relation to the fisher's historical, most-contracted and current range. The historical fisher range occurred prior to European settlement (diagonal hatching), but was reduced to the range at its most contracted state (cross hatching; 43% of the historical range) before expanding to the current range (shaded; 68% of the historical range). White circles represent successful reintroductions or augmentations, black squares represent failed reintroductions, black diamonds represent reintroductions with unknown outcomes, black circles represent ongoing reintroductions and black triangles represent introductions.

**Figure 7:**
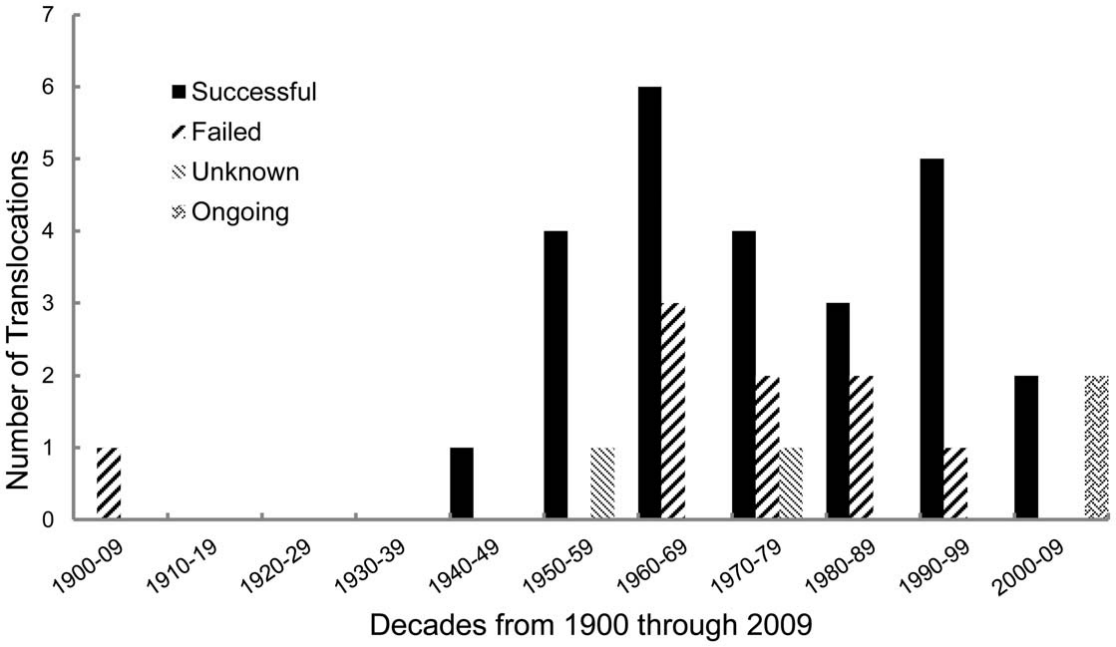
Chronology and success status of 38 fisher translocations.

We documented 38 fisher translocations (30 reintroductions, 5 augmentations, and 3 introductions) in 7 Canadian provinces and 15 US states between 1900 and 2011 (Table 5, Figures 6 and 7). Undocumented translocations also occurred [56]. The first documented fisher translocation in North America was the introduction of 2 fishers to Anticosti Island, Quebec, around 1900 [57]. Fishers were translocated to reestablish a component of the native fauna (63%), to control porcupines (37%), to reestablish a valuable furbearer for commercial trapping (16%), to initiate research (5%), or for combinations of these reasons (26%; Table 5).

**Table 5:** Summary of data for 38 fisher translocations, 1896–2010, listed chronologically.

Release location	Source location	Years(s)	Transloc. type^a^	Number released (♀s)	Success Status^b^	Purpose^c^	References
Quebec	Unknown	1896–1914	I	2 (?)	F	IF	[57]
Nova Scotia	Ranch	1947–1948	R	12 (6)	S	Unknown	[40], [79]
Wisconsin	New York, Minnesota	1956–1963	R	60 (24)	S	PC	[36], [39], [80]–[82]
Ontario	Ontario	1956	R	25 (?)	U	RS	[17], [56]
Ontario	Ontario	1956–1963	R	97 (60)	S	RS	[17], [56]
Montana	British Columbia	1959–1960	A	36 (20)	S	RS,PC,RF	[32], [41], [51], [83], [84]
Vermont	Maine	1959–1967	R	124 (?)	S	PC	[17], [85]
Oregon	British Columbia	1961	R	11 (6)	F	PC	[3], [86], [87]
Oregon	British Columbia	1961	R	13 (8)	F	PC	[3], [86], [87]
Michigan	Minnesota	1961–1963	R	61 (19)	S	PC	[29], [36], [88]
Idaho	British Columbia	1962–1963	A	39 (19)	S	RS,PC	[17], [89], [90], [91]
Nova Scotia	Maine	1963–1966	R	80 (51)	S	RS,PC	[79]
Wisconsin	Minnesota	1966–1967	R	60 (30)	S	PC	[80]–[82]
New Brunswick	New Brunswick	1966–1968	R	25 (15)	S	RS, PC	[43], [92], [93]
West Virginia	New Hampshire	1969	R	23 (?)	S	RS,RF	[42], [94]
Minnesota	Minnesota	1968	R	15 (?)	F	PC	[17], [95]
Maine	Maine	1972	R	7 (3)	U	RS	[17], [96]
Manitoba	Manitoba	1972	R	4 (?)	F	RS	[17], [97]
New York	New York	1976–1979	R	43 (24)	S	RS	[44], [98]
Oregon	British Columbia, Minnesota	1977–1981	R	30 (15)	S	PC	[3]
Colorado	Unknown	1978 or 79	I	2 (1)	F	Unknown	[99]
Ontario	Ontario	1979–1981	R	55 (32)	S	RF	[56], [100], [101]
Ontario	Ontario	1979–1982	R	29 (14)	S	RF	[56], [100], [101]
Alberta	Alberta	1981–1983	R	32 (16)	F	RS	[45], [102], [103]
British Columbia	British Columbia	1984–1991	I	15 (4)	F	PC	[104], [105]
Montana	Minnesota, Wisconsin	1988–1991	R	110 (63)	S	RS	[51], [83]
Michigan	Michigan	1988–1992	R	189 (101)	S	RS,RF	[88]
Connecticut	New Hampshire, Vermont	1989–1990	R	32 (19)	S	RS	[106]–[109]
Alberta	Ontario, Manitoba	1990	R	17 (11)	S	RS,R	[45], [100], [110]
British Columbia	British Columbia	1990–1992	A	15 (13)	S	RS,R	[111]
Nova Scotia	Nova Scotia	1993–1995	A	14 (6)	S	RS	[112]–[115]
Manitoba	Manitoba	1994–1995	R	45 (21)	S	RS	[116]
Pennsylvania	New York, New Hampshire	1994–1998	R	190 (97)	S	RS	[117]
British Columbia	British Columbia	1996–1998	R	60 (36)	F	RS,RF	[66], [67]
Nova Scotia	Nova Scotia	2000–2004	A	28 (21)	S	RS	[113], [114]
Tennessee	Wisconsin	2001–2003	R	40 (20)	S	RS	[68], [118]
Washington	British Columbia	2008–2011	R	90 (50)	O	RS	[50], [119]
California	California	2009–2012	R	40 (24)	O	RS	[120]

Additional data for these 38 translocations are included in Table S1.

a A = augmentation, I = introduction, R = reintroduction.

b F = failure, S = success, U = unknown outcome, O = ongoing.

c IF = introduction of furbearer, PC = porcupine control, RS = reestablish species, RF = reestablish furbearer, R = research.

All 5 augmentations succeeded, whereas 77% (20 of 26) of reintroductions with known outcomes succeeded, and none of the 3 introductions were successful (Table 5). Among reintroductions in eastern North America with known outcomes, 89% (17/19) succeeded, including all that released ≥12 fishers. In contrast, only 43% (3/7) of reintroductions in western North America succeeded; where a release with as few as 17 fishers succeeded but 1 reintroduction of 60 fishers failed. For reintroductions, we obtained data sets for 10 of the 13 variables thought to influence translocation success (Tables 2 and S1). The first 4 variables in Table 2 are, or relate directly to, factors incorporated into our *VORTEX* population model.

**Figure 8:**
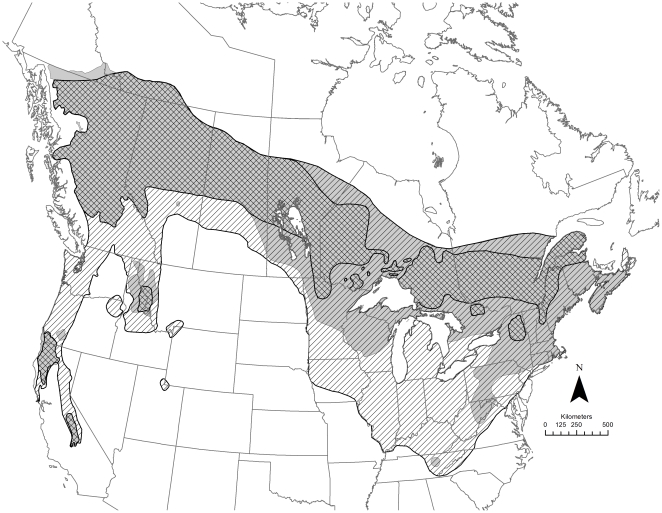
The fisher's historical (diagonal hatching), most-contracted (cross hatching) and current (shaded) ranges. Significant portions of these ranges were obscured by translocation symbols in Figure 6.

Our map of the fisher's geographic distribution (Figure 8) includes the historical range (approximately 5,498,000 km^2^), the most-contracted range (∼2,343,000 km^2^; ∼43% of the historical range) and the current range (∼3,717,000 km^2^; ∼68% of the historical range). The historical range (Figure 8) includes corrections of previous maps by Powell [1] and Gibilisco [18]. Areas where the most-contracted range expanded to form the current range coincide closely with the distribution of successful reintroductions, especially in eastern North America. Several translocation programs used numerous release sites over large areas (e.g., Nova Scotia, Vermont, upper peninsula Michigan, Pennsylvania), and in the case of Nova Scotia, initial reintroductions were followed by augmentations. Fishers were also reintroduced successfully following failed reintroduction attempts in southwestern Oregon and Manitoba.

Range expansion by fishers, however, was not limited to areas where they had been reintroduced. Fishers expanded their range through natural dispersal into areas where they had been extirpated in New York and New England, Ontario and Quebec, Minnesota, and northern California. Fishers also expanded their range naturally into areas where reintroductions failed (e.g., coastal Maine) and into an area outside the historical range in northwestern British Columbia and Yukon Territory (Figures 6 and 8) [54].

### Tests of Predictions from Population Modeling

For actual reintroductions, Number of fishers released, Number of females released and Number of males released were all highly correlated, as expected (*r*>0.97 for all combinations). Number of release sites correlated significantly with Number of males released (*r* = 0.43) and with Number of years that fishers were released (r = 0.47). Number of years that fishers were released also correlated significantly with Number of fishers released, Number of females released, and Number of males released (correlation coefficients 0.56–0.65). We did not combine variables that were significantly correlated in the same hypothesis without blocking to eliminate the effects of the correlation.

When we combined Number of years with Number of fishers, Number of females or Number of males in a hypothesis, we blocked Number of years by the appropriate variable for Number of fishers when calculating likelihoods. We retained 2 single-variable hypotheses from our evaluation of the hypotheses identified in our *VORTEX* population model as influencing reintroduction success (Table 6). The highest ranked hypothesis included only the Number of males released (42% probability of being the most strongly supported hypothesis), but the second-ranked model, including only the Number of females released, was almost as well supported (30% probability). Neither the Number of release sites nor the Number of years were included in any model that we retained, despite our *VORTEX* model simulations identifying the number of release sites as being important.

**Table 6:** Hypotheses generated by the results of our *VORTEX* simulations and their ranking using AIC_C_ 1.

HYPOTHESES	AIC_C_	ΔAIC_C_	*w_i_*
Number Males Released	22.91	0.00	0.415
Number Females Released	23.58	0.67	0.297
Number Females Released, Number Release Sites	26.11	3.20	0.084
Number Females Released, Number Release Years	26.14	3.23	0.083
Number Males Released, Number Release Years	26.14	3.23	0.083
Number Fishers Released	28.94	6.02	0.020
Number Fishers Released, Number Release Sites	31.51	8.59	0.000
Number Fishers Released, Number Release Years	31.53	8.61	0.000
Number Release Sites	52.25	9.34	0.000
Number Release Years	32.28	9.36	0.000

See data for all variables in Table S1.

1 *VORTEX* simulations indicated that the variables in these hypotheses could influence reintroduction success. Only Number of Males and Number of Females were retained for further evaluation (Table 7). In calculating likelihoods, Number of Years was blocked for Number of Fishers, Females or Males released (as appropriate) because these variables were strongly correlated.

We obtained adequate data for 10 additional variables in Table 2 (see full data set in Table S1). We first tested Number of males and Number of females each with Region (eastern vs western North America) and noted that adding Region affected AIC_C_ values profoundly (Table 7). Therefore, we added the other variables one at a time to hypotheses including Region with either Number of females or Number of males (Table 7). The result was that Relatedness of the source animals (indexed by the proximity of source population to the release site; Tables 5 and S1) had the greatest effect on reintroduction success, once Region was already included in the hypothesis (Table 7). Note, however, that all AIC_C_ values in Table 7 are less than those in Table 6, implying that all the hypotheses in Table 7 are able to mimic the original data better than the hypotheses of Number of males and Number of females alone (all hypotheses in Tables 6 and 7 had the same structure and are, therefore, comparable using AIC_C_). The hypotheses including Numbers of males or females with Region and potential Relatedness of the source animals to the original population both have about 45% probabilities of being the hypothesis best able to mimic the data (i.e., best models; Table 7).

**Table 7:** Alternative hypotheses that may affect reintroduction success, and their ranking using AIC_C_.

HYPOTHESES	*AIC_C_*	Δ*AIC_C_*	*w_i_*
Number Males Released, Region, Relatedness	−1.76	0.00	0.447
Number Females Released, Region, Relatedness	−1.76	0.01	0.445
Number Males Released, Region, Diversity	3.48	5.25	0.032
Number Females Released, Region, Diversity	3.52	5.29	0.032
Number Males Released, Region, Type	4.23	5.99	0.022
Number Females Released, Region, Type	4.49	6.26	0.020
Number Females Released, Region, Protect2	7.55	9.31	0.000
Number Males Released, Region, Protect2	7.55	9.32	0.000
Number Females Released, Region	12.38	14.14	0.000
Number Males Released, Region	12.47	14.23	0.000
Number Females Released, Region, Feasibility	13.70	15.47	0.000
Number Females Released, Region, Protect1	13.72	15.49	0.000
Number Males Released, Region, Feasibility	13.92	15.69	0.000
Number Males Released, Region, Protect1	14.04	15.80	0.000
Number Females Released, Region, Season	14.70	16.47	0.000
Number Males Released, Region, Season	14.72	16.49	0.000
Number Males Released, Region, Monitor	15.21	16.98	0.000
Number Females Released, Region, Monitor	15.27	17.03	0.000
Number Males Released, Region, Sex ratio	15.33	17.10	0.000
Number Females Released, Region, Sex ratio	15.33	17.10	0.000

The first two hypotheses are considered the best models. See data for all variables in Table S1.

## Discussion

The fisher is among the most successfully reintroduced carnivores, yet little information is available to explain this success or to guide managers seeking to reestablish fishers. In many areas of the historical range, protection from historical over-trapping may have been all that was needed to prevent extirpation and to maintain a self-sustaining population of fishers. The great success of fisher reintroductions in eastern and mid-western North America in the mid 1900s suggests that the only thing lacking was the fishers themselves, and reintroductions addressed that problem. Past performance, however, does not guarantee future success, and the costs, risks and uncertainties associated with reintroductions prompted us to investigate how we could help managers tilt the odds in their favor. Our results should be helpful as managers continue to reestablish fishers in the vacant areas within the southern portions of the fisher's historical range.

### Increasing Fisher Reintroduction Success

Four factors appear to have a meaningful influence on reintroduction success: the number of females released, number of males released, region (eastern vs. western North America), and the proximity of the source population to the reintroduction area.

It seems intuitively obvious that releasing more females would result in greater success, because releasing more females is expected to result in greater offspring production and greater population growth. Releasing adult females also makes sense, because adult females are sexually mature, and pregnant females can immediately contribute to population growth. Unlike adult females, juvenile females must survive long enough to become sexually mature, successfully mate and give birth, which may not happen for ≥2 years after they are released. The lack of age data for actual reintroductions prevented us from testing the hypothesis that the proportion of adults in a founder population is a meaningful predictor of reintroduction success.

Why the number of males released would have such a meaningful influence on reintroduction success is less obvious to us. Given the non-monogamous mating system of fishers, where a male can mate with >1 female during a breeding season [1], a founder population dominated by females would be more likely to succeed than a founder population of the same size with an even or male-biased sex-ratio. Nonetheless, our analyses indicated that the number of males was at least as important as the number of females for explaining reintroduction success. We believe this result provides important insights about variation in the reproductive abilities of males.

The age, size, and experience of released males is also likely to affect reintroduction success. In other solitary carnivores with large sexual dimorphism in size, extremely large males can dominate smaller males and secure most of the breeding opportunities [58], [59]. Male fishers must survive long enough to reach their maximum body size (at ≥3 years of age), which explains why they represent such a small component of most fisher populations [1]. We hypothesize that the most effective breeding males are large males with pronounced, well-developed musculature on their heads, especially the large temporalis muscles that originate along the sides of the skull and attach to their large sagittal crests [1], [60], [61]. These muscles enlarge during the breeding season and shrink during summer, suggesting a relationship with breeding (Powell unpublished data). We hypothesize that such males are the most effective breeders and the strongest competitors for reproductive females, and as such, they would make more significant contributions to translocation success than young males. If a reproductive advantage for large and experienced males exists in translocated fisher populations, then relatively few large males need to be released to achieve reintroduction success. Reintroduction programs that released larger numbers of fishers were likely to include a greater number of these large breeding males, thereby increasing the likelihood of success.

We hypothesize that for other mammals where reproduction of males has a large skew, reintroduction success should depend on the number of males that are effective breeders and not on the total number of males released, as we hypothesize for fishers. Males of many mammals exhibit sexually dimorphic, sexually selected traits that could be the basis for such reproductive skew [e.g. 58], [ 59]. We encourage biologists who plan reintroductions of such mammals to investigate the logistics of selecting males for release based on their sexually dimorphic traits and determine how those traits affect reproductive success.

We can not explain the difference in success of reintroductions in eastern *vs.* western North America. Our data, the information available in the literature, and personal communications from agency personnel involved in reintroductions provide no insights into why reintroductions in the East have twice the probability of success as those in the West. Differences in snow cover [62], forest succession, and forest characteristics [63] have been proposed as contributing factors. Forestry practices, predator-prey communities, and genetic characteristics also differ by region and may play a role.

Our analyses of actual reintroductions suggest that releasing fishers from a nearby source population (a population from the same part of the continent) positively affects the probability of reintroduction success (Table 7). Many successful reintroductions used source populations from the same or nearby states and provinces. This result suggests that some local, genetic adaptation may exist within fisher populations.

Releasing animals into suboptimal habitat is a major reason for failure of translocations in general [8], [64], [65]. We assume that project managers released fishers into areas they considered to be suitable habitat, yet only 4 reintroduction projects conducted formal assessments of habitat quality prior to initiating releases (southeastern British Columbia [66], [67]; Tennessee [68], Washington [69], [70] and California [15]). Consequently, we lack data for one of the most significant factors affecting success (habitat adequacy) for 89% (34 of 38) of documented fisher translocations. Our small sample size of translocations with formal habitat assessments was too small to draw meaningful conclusions from the data, however, the value of a formal habitat assessment seems clear given the uncertainties and the resources at stake.

The 100% success rate for the 5 fisher augmentations suggests that they are more likely to be successful than reintroductions (77% success). While there are several reasons why augmentations could have a greater likelihood of success than reintroductions, the indications of success are also less clear for augmentations. Continued persistence of a small, reestablished population can demonstrate the success of a reintroduction, whereas some indication of population growth, expansion or improvement is necessary to conclude that an augmentation was successful. The presence of a small resident population of fishers could easily increase the likelihood of success for augmentations, as these resident fishers may serve as a locally adapted foundation for population expansion that does not exist where reintroductions are initiated. Augmentations may also remedy a skewed sex-ratio that limited population growth in a small resident population or add new genotypes to a population with low genetic diversity. The data for augmentations were also complicated by fisher releases in Montana (1959–1960) and Idaho (1962–1963), which were thought to be reintroductions because, at the time, no one knew that a remnant, native population still existed in the Rocky Mountains of eastern Idaho and western Montana [32], [33]. We do not know if managers would have hesitated to release new fishers in this area had they known of the remnant native population. Nor do we know if these augmentations prevented the loss of the remnant native population or if they influenced fisher fitness. What is clear, however, is that the fisher range expanded from its most-contracted state in this region following the augmentations.

We documented 3 fisher introductions, and while each failed, each was unique in its circumstances. An introduction of 2 fishers to Anticosti Island, Quebec was conducted around 1900 by the Island's owner, Henry Menier, whose goal was to introduce a number of game species to the Island for future harvest [57]. The second introduction involved the captive-rearing and release of 2 fishers (male and female siblings) in west-central Colorado around 1978 by Marty Stouffer as part of his “Wild America” television series (season 3, Fishers in the family, episodes 8 and 9; www.wildamerica.com). Stouffer's purpose for releasing the fishers may have been solely to create an interesting television show while evicting an unruly duo of fishers from his home. The third introduction was conducted from 1984 to 1991 in an area just outside the historical range in northern-coastal British Columbia; 11 males and 4 females (2 of the 4 were badly injured) were released to control porcupines. While we have almost no information to speculate on why these introductions failed or the suitability of habitat at the release sites, the success of an introduction involving the release of only 2 fishers or only 2 uninjured females would be extremely unlikely.

Reintroduction success did not differ for hard *vs* soft releases in our analyses, even though all soft releases were successful. Our test lacked power, however, because the number of soft releases was very small (n = 3). Similarly, our analyses failed to detect any effects of protecting released fishers from trapping or of releasing fishers in different seasons. State and provincial regulations related to protection were diverse and difficult to categorize, and likely prevented us from detecting the true effects of no protection. Moreover, some reintroduced fishers may have benefited from informal protection from trappers that wanted reintroductions to succeed. In addition, our analyses could not detect effects of post-release monitoring on reintroduction success. An evaluation of monitoring effects was complicated by the lack of formal monitoring efforts in the earlier fisher translocations, and agency reports and publications consistently failed to mention management actions that could have been triggered by monitoring results to increase the likelihood of success.

Our simulations suggest that the number of years over which a targeted number of fishers are released should not affect the probability of success. Nonetheless, among actual reintroductions, successful reintroductions involved releasing fishers over multiple years, probably because logistical and financial constraints limit the number of fishers that can be obtained and released in a given year. For example, a reintroduction project could target 100 fishers for release, but limitations on trapping conditions (e.g., suitable weather, adequate road access) and trapping success may require trapping over a number of years to reach the target number of animals to release. Thus, despite evidence to the contrary, releasing fishers over multiple years may be required out of practical necessity.

Our simulations and analyses focused on fisher populations, but the factors that influence fisher reintroduction success are also likely to influence the reintroduction success of other species, including other mustelids, other carnivores, and other wide-ranging mammals that do not live in groups or have other complex social organizations. Consistent with our *VORTEX* simulations, we expect that the number of adult females released, the sex-ratio within a founder population, and the number of release sites to be factors that are likely to influence carnivore reintroduction success. Our analyses of data from actual reintroductions suggest that the number of males released and the proximity of the source population could also be meaningful predictors of carnivore reintroduction success. While some factors that influence reintroduction success are likely to be species- or area-specific, we expect one or more of the factors we have identified to be informative to managers as they plan and implement carnivore reintroductions and that this information will improve their likelihood of success.

### The Fisher's Range, Translocations and Conservation

While strict harvest regulations in many states and provinces provided significant protection for fishers from overexploitation across much of their historical range, no one has conducted a range-wide evaluation of how reintroductions and augmentations have contributed to fisher conservation. Our depictions of the historical, most-contracted and current fisher ranges illustrate the vulnerability of this carnivore to a variety of threats [71]; they also provide meaningful baselines for measuring recovery. The expansion of the most-contracted range to the current range was most extensive in the eastern and Great Lake states and provinces. For a number of these states and provinces, range expansions may have resulted from successful reintroductions, natural range expansions, or both (e.g., Maine, New Brunswick, New York, New Hampshire, Ontario, Vermont). Reintroductions, however, were responsible for the reestablishment of fisher populations in Maryland, Michigan, Nova Scotia, Pennsylvania, West Virginia, and Tennessee, long before a natural range expansion could have occurred.

Given the success of individual reintroductions and the range-wide consequences of successful reintroductions, it is clear that reintroductions have made a significant contribution to fisher conservation. Much of the fisher's recovery was the result of uncoordinated reintroduction programs conducted within individual states and provinces that occurred long before the fisher was a candidate for federal listing. However, the recovery of the fisher suggests that if uncoordinated restoration efforts can result in a significant range expansion of a wide-ranging carnivore, coordinated and well-planned restoration efforts could easily result in a similar or greater level of success.

### Management Recommendations

Because the number of females and males released is critical to reintroduction success, we recommend that managers release as many fishers as possible. We expect the likelihood of success to be improved by acquiring a founder population that is slightly female-biased (e.g., 55–60% females), and one that is also adult-biased, in an effort to obtain a greater proportion of pregnant females and large males that are effective breeders. Although we are not in a position to recommend a minimum number of fishers to release, we observed that 9 of 10 reintroductions that released ≥60 fishers were successful, and all 4 reintroductions of ≥100 fishers were successful. (We also note that all reintroductions in eastern North America with ≥12 fishers succeeded.)

We recommend that managers select source populations that are close to release sites. Using multiple release sites and diverse source populations may also increase the probability of success. Given the fisher's vulnerability to overharvest and incidental capture [1], protection from direct and incidental harvest should improve the likelihood of reintroduction success, even though our analyses could not verify this effect. While a formal feasibility assessment is recommended for any translocation [10], [12], only 5 fisher translocations were preceded by formal assessments; all 5 were among the most recent translocations (1994–2011). Feasibility assessments are essential given the cost, uncertain outcome, and risk to source populations associated with translocations, especially for species at risk. Although a feasibility assessment is useful for evaluating the likelihood that a reintroduction will succeed, an assessment also provides the foundation for a reintroduction implementation plan [15], [69], [70]. These and other planning efforts (e.g., species status reviews, recovery planning, NEPA analyses) can help managers minimize or avoid controversies associated with reintroductions and, thereby, increase support for reintroduction projects.

Future reintroductions should incorporate active, post-release monitoring with effective adaptive management [10]. Passive monitoring alone (e.g., incidental observations, road-kill mortalities) is insufficient to assess translocation success and to identify avoidable hazards. Reintroductions and monitoring programs are expensive and essential funding may be contingent upon progress demonstrated through monitoring. However, monitoring efforts can be coordinated with research programs, saving time and money.

With growing concern for species persistence in the face of climate change [72]–[75], wildlife managers and researchers have had to consider new approaches to protecting species at risk. Assisted colonization, (aka, managed relocation) is the translocation of individuals of a species at risk of extinction to a location outside their historical range, where their likelihood of persistence is greater [76]–[78]. While assisted colonization could be essential for protecting a species whose habitats are disappearing as a result of climate change, it is also controversial in that it could result in a harmful invasion of a species into habitats critical to other species. The status of the fisher may not decline to the point where assisted colonization is required to protect the species from extinction, yet, the factors that we have identified that influence reintroduction success would likely influence the success of assisted colonizations of fishers and other at-risk species.

## Supporting Information

Table S1 **The complete set of attribute data used in our analyses of 38 fisher translocations, listed chronologically.** References in this table refer to sources listed in the reference section of the manuscript.
(DOC)

Program File S1 **A program file created for our** ***VORTEX*** **simulations is included as supplemental information to interested readers (Program File S1.vpj).**
(VPJ)
