# Supplementary material for: Carnivore Translocations and Conservation: Insights from Population Models and Field Data for Fishers (Martes pennanti)
Source: PLoS One. 2012 Mar 27;7(3):e32726. doi: 10.1371/journal.pone.0032726 (PMC3314015; doi:10.1371/journal.pone.0032726)
Supplement: Table S1 — The complete set of attribute data used in our analyses of 38 fisher translocations, listed chronologically. References in this table refer to sources listed in the reference section of the manuscript. (DOC) [file pone.0032726.s001.doc]

Table S1. The complete set of attribute data used in our analyses of 38 fisher translocations, listed chronologically. References in this table refer to sources listed in the reference section of the manuscript.

| Release location | Source location | Years(s) | Translocation Type^a^ | Number released (♀s) | Number of release locations | Success Status^b^ | Purpose^c^ | Release season(s) | Region of North America^d^ | Release type^e^ | Proximity of source population | Feasibility study pre-release | Formal post-release monitoring | Protection via closed fisher trapping season | Protection from incidental  capture | References |
| --- | --- | --- | --- | --- | --- | --- | --- | --- | --- | --- | --- | --- | --- | --- | --- | --- |
| Quebec | Unknown | 1896-1914 | I | 2 (?) | Unknown | F | IF | Unknown | Eastern | Unknown | Unknown | No | No | No | No | 56 |
| Nova Scotia | Ranch | 1947-1948 | R | 12 (6) | Unknown | S | Unknown | Su | Eastern | Hard | Unknown | No | No | Unknown | No | 40,78 |
| Wisconsin | New York, Minnesota | 1956-1963 | R | 60 (24) | 1 | S | PC | W | Eastern | Hard | Near and Far | No | Yes | Yes | Yes | 36,39,79-81 |
| Ontario | Ontario | 1956 | R | 25 (?) | Unknown | U | RS | Unknown | Eastern | Hard | Near | No | Yes | Yes | No | 17,82 |
| Ontario | Ontario | 1956-1963 | R | 97 (60) | Unknown | S | RS | Unknown | Eastern | Hard | Near | No | Yes | Yes | No | 17,82 |
| Montana | British Columbia | 1959-1960 | A | 36 (20) | 3 | S | RS, PC, RF | W, Sp | Western | Hard | Near | No | No | Yes | Yes | 32,41,51,83,84 |
| Vermont | Maine | 1959-1967 | R | 124 (?) | 35 | S | PC | Sp | Eastern | Hard | Near | No | No | Yes | No | 17,85 |
| Oregon | British Columbia | 1961 | R | 11 (6) | 1 | F | PC | W | Western | Hard | Near | No | No | Yes | Yes | 3,86,87 |
| Oregon | British Columbia | 1961 | R | 13 (8) | 1 | F | PC | Sp | Western | Hard | Near | No | No | Yes | Yes | 3,86,87 |
| Michigan | Minnesota | 1961-1963 | R | 61 (19) | 1 | S | PC | F or W | Eastern | Unknown | Near | No | No | Yes | Unknown | 29,36,88 |
| Idaho | British Columbia | 1962-1963 | A | 39 (19) | 3 | S | RS, PC | W | Western | Hard | Near | No | No | Yes | No | 17,89,90,91 |
| Nova Scotia | Maine | 1963-1966 | R | 80 (51) | 8 | S | RS, PC | Unknown | Eastern | Unknown | Unknown | No | No |  | No | 78 |
| Wisconsin | Minnesota | 1966-1967 | R | 60 (30) | 1 | S | PC | F, W, Sp | Eastern | Hard | Near | No | No | Yes | Yes | 79-81 |
| New Brunswick | New Brunswick | 1966-1968 | R | 25 (15) | 2 | S | RS, PC | W, Sp | Eastern | Hard | Near | No | No | Yes | No | 43,92,93 |
| West Virginia | New Hampshire | 1969 | R | 23 (?) | 2 | S | RS, RF | W | Eastern | Hard | Near | No | No | No | No | 42,94 |
| Minnesota | Minnesota | 1968 | R | 15 (?) | 1 | F | PC | F, W | Eastern | Hard | Near | No | No | Yes | Yes | 17,95 |
| Maine | Maine | 1972 | R | 7 (3) | 1 | U | RS | W | Eastern | Unknown | Near | No | Unknown | No | No | 17,96 |
| Manitoba | Manitoba | 1972 | R | 4 (?) | 1 | F | RS | W | Eastern | Hard | Near | No | No | Yes | Yes | 17,97 |
| New York | New York | 1976-1979 | R | 43 (24) | 1 | S | RS | F | Eastern | Hard | Near | No | Yes | Yes | No | 44,98 |
| Oregon | British Columbia, Minnesota | 1977-1981 | R | 30 (15) | 1 | S | PC | F, W, Sp | Western | Hard | Far | No | No | Yes | No | 3 |
| Colorado | Unknown | 1978 or 1979 | I | 2 (1) | 1 | F | Unknown | Unknown | Western | Hard | Unknown | No | No | Yes | No | 99 |
| Ontario | Ontario | 1979-1981 | R | 55 (32) | 1 | S | RF | F, W | Eastern | Hard | Near | No | Yes | Yes | No | 82,100,101 |
| Ontario | Ontario | 1979-1982 | R | 29 (14) | 1 | S | RF | F, W | Eastern | Hard | Near | No | Yes | Yes | No | 82,100,101 |
| Alberta | Alberta | 1981-1983 | R | 32 (16) | 1 | F | RS | F | Western | Hard | Near | No | Yes | Yes | No | 45,102,103 |
| British Columbia | British Columbia | 1984-1991 | I | 15 (4) | 1 | F | PC | W, Su | Western | Hard | Near | No | Yes | No | No | 104,105 |
| Montana | Minnesota, Wisconsin | 1988-1991 | R | 110 (63) | 1 | S | RS | F, W | Western | Hard and Soft | Far | No | Yes | No | No | 51,83 |
| Michigan | Michigan | 1988-1992 | R | 189 (101) | 9 | S | RS, RF | W | Eastern | Unknown | Unknown | No | No | No | No | 88 |
| Connecticut | New Hampshire, Vermont | 1989-1990 | R | 32 (19) | 1 | S | RS | W | Eastern | Hard and Soft | Near | No | Yes | Yes | No | 106-109 |
| Alberta | Ontario, Manitoba | 1990 | R | 17 (11) | 1 | S | RS, R | W, Su | Western | Soft | Far | No | Yes | Yes | Yes | 45,100 |
| British Columbia | British Columbia | 1990-1992 | A | 15 (13) | 1 | S | RS, R | F, W | Western | Soft | Near | No | Yes | No | No | 111 |
| Nova Scotia | Nova Scotia | 1993-1995 | A | 14 (6) | 1 | S | RS | W | Eastern | Hard | Near | No | No | Yes | No | 112-115 |
| Manitoba | Manitoba | 1994-1995 | R | 45 (21) | 1 | S | RS | Su, F | Eastern | Hard | Near | Yes | Yes | Yes | Yes | 116 |
| Pennsylvania | New York, New Hampshire | 1994-1998 | R | 190 (97) | 6 | S | RS | F, Sp, Su | Eastern | Hard | Near | Yes | Yes | Yes | No | 117 |
| British Columbia | British Columbia | 1996-1998 | R | 60 (36) | 1 | F | RS, RF | Sp, Su | Western | Hard | Near | Yes | Yes | No | No | 65,66 |
| Nova Scotia | Nova Scotia | 2000-2004 | A | 28 (21) | 1 | S | RS | Sp, Su, Unknown | Eastern | Hard | Near | No | Yes | Yes | No | 113,114 |
| Tennessee | Wisconsin | 2001-2003 | R | 40 (20) | 1 | S | RS | F | Eastern | Hard | Near | No | Yes | Yes | No | 67,118 |
| Washington | British Columbia | 2008-2011 | R | 90 (50) | 9 | O | RS | W | Western | Hard | Near | Yes | Yes | Yes | Yes | 50,119 |
| California | California | 2009-2012 | R | 15 (9) | 1 | O | RS | W | Western | Hard | Near | Yes | Yes | Yes | Yes | 120 |
